# Supplementary material for: ProteinShader: illustrative rendering of macromolecules
Source: BMC Struct Biol. 2009 Mar 30;9:19. doi: 10.1186/1472-6807-9-19 (PMC2672931; doi:10.1186/1472-6807-9-19)
Supplement: Additional file 1 — ProteinShader program without source code. This compressed file contains the complete ProteinShader program including associated libraries, but no source code. A README.txt file gives an overview of the ProteinShader distribution, and the index.html file in the help subdirectory has directions on getting started with the program as well as a set of tutorials. [file 1472-6807-9-19-S1.zip › ProteinShader-beta-0_9_4-binary/help/api/org/proteinshader/structure/Record.html]

Record (ProteinShader API)


|  |  |  |
| --- | --- | --- |
| **Overview** \| **Package** \| **Class** \| **Use** \| **Tree** \| **Deprecated** \| **Index** \| **Help** | |  |
| **PREV CLASS**   **NEXT CLASS** | **FRAMES**    **NO FRAMES**     **All Classes** |  |
| SUMMARY: NESTED \| FIELD \| CONSTR \| METHOD | DETAIL: FIELD \| CONSTR \| METHOD |  |


---


## org.proteinshader.structure Class Record

```
java.lang.Object
  org.proteinshader.structure.Record
```

---

``` public class Record extends Object ```

A Record object serves as a container for the lines of a PDB record.

---

**Constructor Summary**

`Record(String categoryName,
String recordName)`
  
          Constructs a Record with the name specified as an argument.


| **Method Summary** | |
| --- | --- |
| `void` | `addLine(String line)`             Adds a line to the Record. |
| `void` | `clear()`             Clears all lines from the Record. |
| `String` | `getCategoryName()`             Returns the name of the Category the Record belongs to. |
| `String` | `getName()`             Returns the name of the Record. |
| `Iterator<String>` | `iteratorLines()`             Returns a Iterator for the lines of the Record. |
| `int` | `numberOfLines()`             Returns the number of lines in the Record. |
| `void` | `removeLine(String line)`             Removes the first occurrence of a line from the Record. |

**Methods inherited from class java.lang.Object**

`clone, equals, finalize, getClass, hashCode, notify, notifyAll, toString, wait, wait, wait`

**Constructor Detail**

### Record

```
public Record(String categoryName,
              String recordName)
       throws InvalidIDException
```

:   Constructs a Record with the name specified as an argument.
    An InvalidIDException will be thrown if the record name does not
    have at least one character. The categoryName is not checked
    because it should have already been checked by the constructor
    of the Category object that creates and owns this Record.

    **Parameters:**: `recordName` - the name of the Record.: `categoryName` - the name of the Category. **Throws:**: `InvalidIDException` - if recordName is null or an empty String.


**Method Detail**

### getName

```
public String getName()
```

:   Returns the name of the Record.
    The Record name cannot be null or an empty String because is
    checked by the constructor and is read-only.

    :   **Returns:**: The Record name.

---


### getCategoryName

```
public String getCategoryName()
```

:   Returns the name of the Category the Record belongs to.

    :   **Returns:**: The Category name.

---


### addLine

```
public void addLine(String line)
```

:   Adds a line to the Record.
    In a PDB formatted file the lines will never exceed 80 characters,
    which will make them easy to present in a GUI text area. If line
    is null, nothing is added to the Record (and no exception is
    thrown).

    :   **Parameters:**: `line` - a line of the Record.

---


### iteratorLines

```
public Iterator<String> iteratorLines()
```

:   Returns a Iterator for the lines of the Record.
    The iteration order will be the same as the order in which the
    lines were added to the Record. Because the lines are from a PDB
    record, they should never be longer than 80 characters.

    :   **Returns:**: An Iterator for the lines of the Record.

---


### numberOfLines

```
public int numberOfLines()
```

:   Returns the number of lines in the Record.

    :   **Returns:**: The number of lines in the Record.

---


### removeLine

```
public void removeLine(String line)
```

:   Removes the first occurrence of a line from the Record.

    :   **Parameters:**: `line` - the line to remove.

---


### clear

```
public void clear()
```

:   Clears all lines from the Record.


---


|  |  |  |
| --- | --- | --- |
| **Overview** \| **Package** \| **Class** \| **Use** \| **Tree** \| **Deprecated** \| **Index** \| **Help** | |  |
| **PREV CLASS**   **NEXT CLASS** | **FRAMES**    **NO FRAMES**     **All Classes** |  |
| SUMMARY: NESTED \| FIELD \| CONSTR \| METHOD | DETAIL: FIELD \| CONSTR \| METHOD |  |


---

# *Copyright © 2007-2008*
